# Role of lymphocyte-to-C-reactive protein ratio in forecasting microvascular obstruction in ST-segment elevation myocardial infarction post-percutaneous coronary intervention

**DOI:** 10.3389/fcvm.2025.1526057

**Published:** 2025-03-26

**Authors:** Jiahua Liu, Xinjia Du, Yanfei Ren, Yan Mei, Lei Chen, Yuan Lu

**Affiliations:** ^1^ Department of Cardiology, The Affiliated Hospital of Xuzhou Medical University, Xuzhou, China; ^2^ Department of Cardiology, Shanghai Tenth People’s Hospital, Tongji University School of Medicine, Shanghai, China

**Keywords:** lymphocyte-to-C-reactive protein ratio, microvascular obstruction, ST-segment elevation, myocardial infarction, percutaneous coronary intervention

## Abstract

**Background:**

Current research suggests that microvascular obstruction (MVO) following the first percutaneous coronary intervention (PCI) in myocardial infarction patients is closely related to inflammatory responses. The lymphocyte-to-C-reactive protein (CRP) ratio (LCR), as a novel inflammatory marker, is associated with the prognosis of patients with ST-segment elevation myocardial infarction (STEMI). However, the relationship between LCR and MVO remains unclear. This study aims to investigate the correlation between LCR and MVO in STEMI patients undergoing PCI.

**Methods:**

This was a single-center retrospective study. We consecutively enrolled STEMI patients who underwent PCI at Xuzhou Medical University Affiliated Hospital, Xuzhou, China, from September 2019 to December 2023. Cardiac magnetic resonance (CMR) imaging with late gadolinium enhancement (LGE) was used to assess infarct size and the presence of MVO.

**Results:**

A total of 551 patients were included in this study, with 267 (48.5%) experiencing MVO. The median time for CMR imaging-based detection of MVO was 5 days (interquartile range: 4, 6). Univariate regression analysis revealed that age, white blood cell count, neutrophil count, left ventricular ejection fraction (LVEF), peak N-terminal pro-B-type natriuretic peptide (NT-proBNP), peak high-sensitivity troponin T (hs-TnT), LCR, LGE percentage (LGE%), and MVO percentage (MVO%) were significantly associated with MVO (*p* < 0.05). Multivariate regression analysis identified LCR as an independent predictor of MVO [Odds Ratio = 0.18, 95% Confidence Interval (CI): 0.04–0.75, *p* = 0.019]. Receiver operating characteristic curve analysis demonstrated that LCR had predictive capability for MVO, with a sensitivity of 80.1% and specificity of 45.4% when the LCR value was 0.091 [area under the curve (AUC): 0.654, 95% CI: 0.609–0.700, *p* < 0.001]. A new predictive model incorporating LCR improved the prediction of MVO occurrence (AUC = 0.815, *p* < 0.001), with significant differences in net reclassification improvement (*p* = 0.004) and integrated discrimination improvement (*p* = 0.023) between the new and old models.

**Conclusion:**

A low LCR is an independent risk factor for MVO after PCI in STEMI patients. The predictive model incorporating LCR enhances the ability to predict MVO occurrence in patients with STEMI post-PCI.

## Introduction

ST-segment elevation myocardial infarction (STEMI) is caused by plaque rupture and complete thrombotic occlusion of the coronary artery, leading to insufficient blood supply and necrosis in the affected myocardial region (1). As one of the most severe types of cardiovascular diseases, it poses a significant threat to the health and survival of the patient. Although advancements have been made in reperfusion therapies such as percutaneous coronary intervention (PCI) and thrombolysis, the mortality rate of patients with STEMI remains high. While PCI can resolve blockages in major vessels, microvascular damage cannot be effectively addressed through current surgical methods. Research has shown that 50% of patients still experience microvascular obstruction (MVO) after PCI (2), and this is a key factor affecting the prognosis of patients with STEMI (3). Therefore, identifying simple and reliable biomarkers to detect microcirculatory disorders is of great clinical importance.

The lymphocyte-to-C-reactive protein ratio (LCR) is a novel inflammatory biomarker that reflects the balance between systemic inflammatory and immune responses. Current research suggests that LCR can be used to assess prognosis in patients with malignant tumors (4), indicate the progression of coronavirus disease 2019 (COVID-19) (5), and predict adverse outcomes in patients with hepatitis B virus-related decompensated cirrhosis (6). In cardiovascular diseases, LCR has been recognized as a predictor of adverse cardiovascular events in patients with STEMI post-PCI and as a risk factor for infection-related mortality following cardiac surgery (7, 8). Additionally, LCR has shown value in predicting subclinical myocardial injury in the general male population and is an independent predictor of new-onset atrial fibrillation in patients with myocardial infarction post-PCI (9, 10).

During the pathophysiological process of STEMI, inflammatory responses play a crucial role in plaque rupture and also affect myocardial reperfusion and recovery. The occurrence of MVO is closely associated with inflammation, thrombus burden, and endothelial dysfunction (2). Given that LCR reflects the systemic inflammatory response, it remains unclear whether it is related to the occurrence of MVO. This study aimed to explore the potential relationship between LCR and MVO in patients with STEMI post-PCI, providing new insights for the early identification of high-risk patients in clinical practice.

## Methods

This study was a single-center, retrospective investigation. Patients with STEMI who were consecutively admitted and underwent PCI within 12 h of admission at Xuzhou Medical University Affiliated Hospital, Xuzhou, China between September 2019 and December 2023 were included in this study. STEMI was diagnosed based on European Society of Cardiology guidelines, characterized by ST-segment elevation (≥1 mm in at least two contiguous leads on the electrocardiogram) and elevated serum levels of myocardial injury biomarkers [e.g., high-sensitivity troponin T (hs-TNT)], confirmed by coronary angiography showing arterial occlusion (11).

**Inclusion criteria**: All enrolled patients who underwent successful PCI [post-PCI Thrombolysis in Myocardial Infarction (TIMI) flow grade ≥ 2] within 12 h of presenting with symptoms and were examined via cardiac magnetic resonance (CMR) imaging during hospitalization were included.

**Exclusion criteria**: Patients with a prior history of myocardial infarction, autoimmune diseases, infection-related illnesses (e.g., bacterial pneumonia, tuberculosis, viral infections such as influenza or COVID-19), malignant tumors, hematologic diseases, or poor-quality CMR images were excluded.

**Figure 1:**
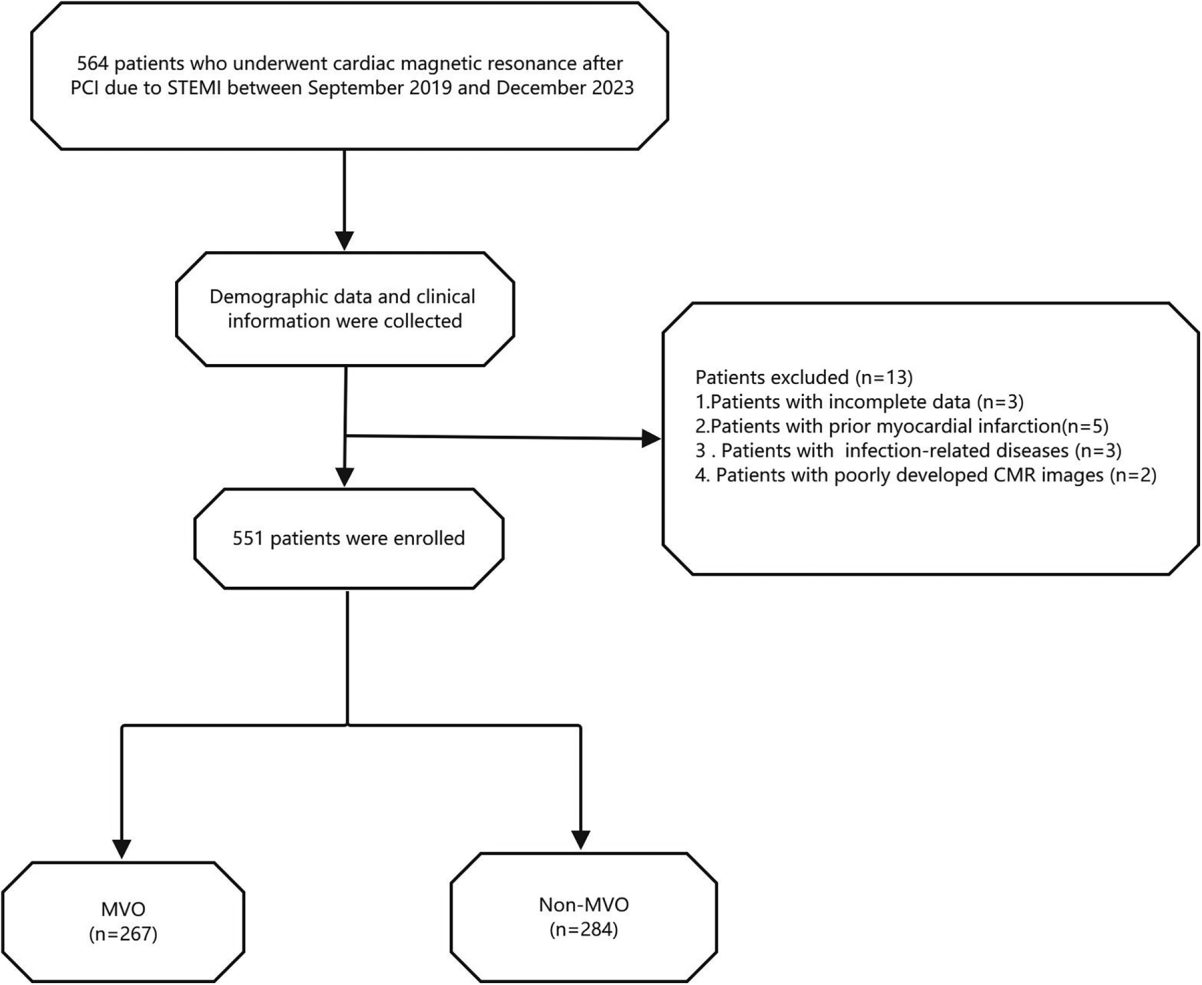
Flow chart of the study population. STEMI, ST-segment elevation myocardial infarction; MVO, microvascular obstruction.

This study was approved by the Ethics Committee of Xuzhou Medical University Affiliated Hospital, Xuzhou, China. Based on the relevant Institutional Review Board regulations, and because this study was retrospective and posed no risk to patients, the requirement for written informed consent was waived (Ethics Approval Number: XYFY2024-KL207-01). The inclusion and exclusion criteria are shown in Figure 1.

**Inflammatory and clinical indicators**: We collected baseline patient information, including name, height, weight, age, smoking history, history of hypertension and diabetes, systolic blood pressure, diastolic blood pressure, heart rate, length of hospital stay, timing of post-PCI CMR examination, and Killip class. Routine blood tests were performed during hospitalization, and data on white blood cells, lymphocytes, neutrophils, platelets, high-sensitivity C-reactive protein (hs-CRP), troponin, N-terminal pro-B-type natriuretic peptide (NT-proBNP), blood lipids, glycated hemoglobin, and glomerular filtration rate were collected. Notably, white blood cell, lymphocyte, and neutrophil counts were collected preoperatively, while hs-CRP, hs-TNT, and NT-proBNP levels represented the peak values measured during hospitalization. We also collected preoperative TIMI flow grade values and infarct-related artery data based on coronary angiography findings (classified as TIMI ≤ 1).

**Figure 2:**
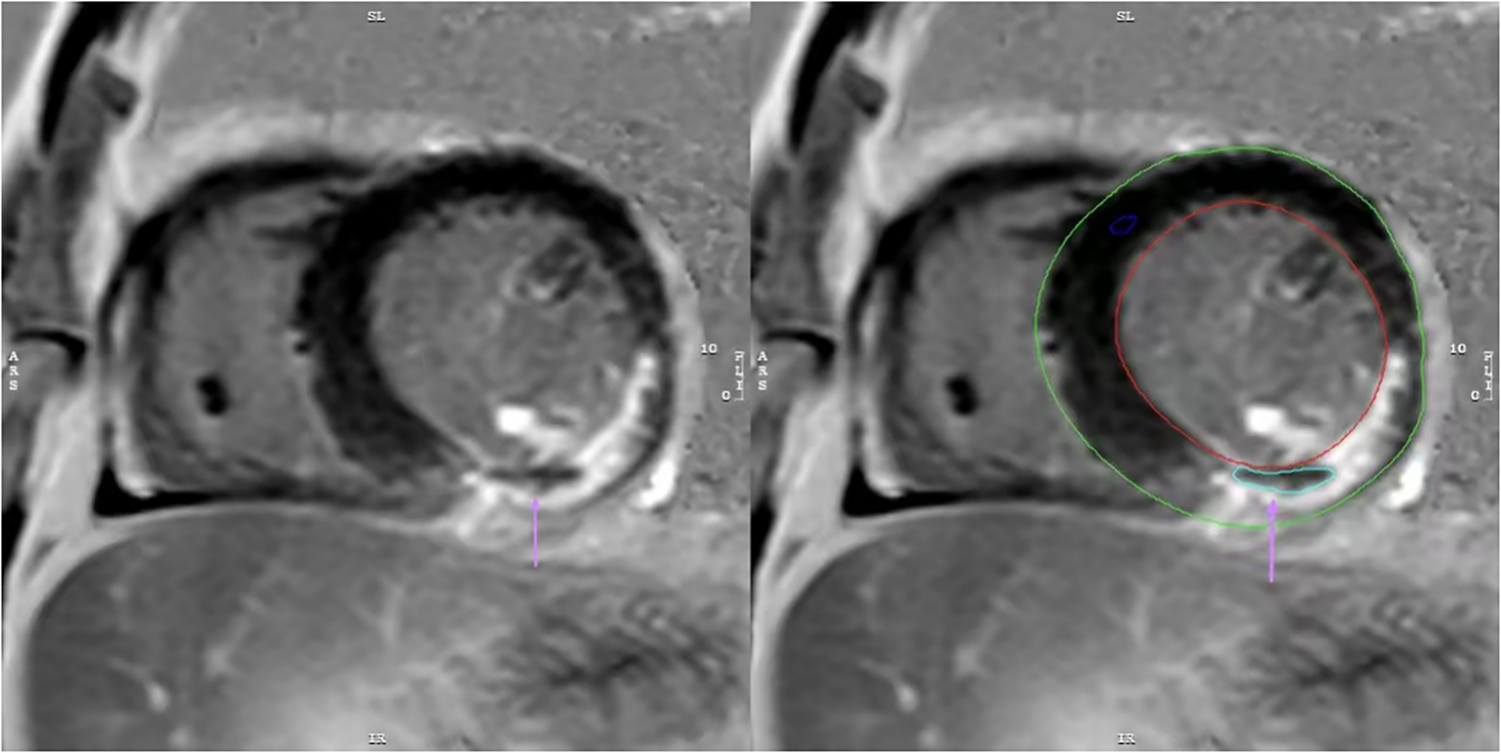
The image depicts a patient with myocardial infarction on the LGE sequence, manually outlined using CVI42 software. MVO is present as a region of low signal within the infarct area(↑).

**CMR and MVO image analysis**: CMR images were acquired using a 3.0 T magnetic resonance scanner (Ingenia 3.0 T, Philips, Netherlands) during hospitalization, with a median timing of 5 days (interquartile range: 4–6 days). Images were acquired using the late gadolinium enhancement (LGE) sequence. Post-processing software (Cvi42, version 5.13.5, Circle V Vascular Imaging, Canada) was used to quantify cardiac parameters from CMR images. MVO was defined as low-signal areas surrounded by high-signal myocardial tissue in LGE images, and its mass was manually quantified relative to total myocardial mass (expressed as MVO%, Figure 2) (12). Similarly, LGE was defined as the myocardial infarct size percentage and was quantified as the ratio of hyper-enhanced myocardial mass relative to total myocardial mass (expressed as LGE%). A diagnosis of MVO was confirmed by experienced cardiovascular physicians, who were blind to related clinical data of patients to avoid bias, and all CMR data interpreted by them.

**Statistical Analysis**: The Kolmogorov–Smirnov test was used to assess the normality of data distribution. Normally distributed continuous variables were expressed as mean ± standard deviation (*x̅* ± *s*) and analyzed using the independent samples *t*-test. Nonnormally distributed data were presented as medians and quartiles and analyzed using the Mann–Whitney *U* test. Categorical data were analyzed using the chi-square test. All indicators were grouped based on the presence or absence of MVO, and differences between groups were analyzed. The relationship between LCR levels and MVO% was assessed for linearity using Pearson's correlation coefficient. To avoid multicollinearity, two variables involved in the calculation of LCR were excluded, and the remaining variables were included in the univariate logistic regression analysis. Variables with *p*-values < 0.05 in univariate logistic regression analysis were included in multivariate stepwise logistic regression to identify independent risk factors for MVO after PCI in patients with STEMI. The diagnostic value of LCR for MVO in patients with STEMI post-PCI was evaluated using receiver operating characteristic (ROC) curves. The newly identified independent risk factors were incorporated into a new predictive model, and net reclassification improvement (NRI) and integrated discrimination improvement (IDI) were calculated to assess the predictive performance of the model compared to the old one. Statistical analysis was performed using SPSS version 27.0 and R version 4.3.1, with graphs generated using GraphPad Prism version 9. All statistical analyses were two-tailed, with *p*-values < 0.05 considered statistically significant.

## Results

### Baseline data comparison between groups

Among the 551 patients who underwent CMR imaging during hospitalization, 267 patients (48.5%) were diagnosed with MVO and 284 patients (51.5%) exhibited no MVO. The baseline characteristics of all patients are shown in Table 1. Compared to patients without MVO, those with MVO were older and had higher peak levels of hs-CRP, NT-proBNP, and hs-TNT, as well as higher white blood cell and neutrophil counts, and a larger LGE percentage. They also had longer hospital stays, lower left ventricular ejection fraction (LVEF), lymphocyte counts, neutrophil-to-lymphocyte ratio (NLR) and LCR levels, with statistically significant differences (*P* < 0.01). Preoperative TIMI flow grade (TIMI ≤ 1) was also significantly different between the two groups (*P* < 0.001).

**Table 1:** Patient characteristics.

Variables	Total (*n* = 551)	Non-MVO (*n* = 284)	MVO (*n* = 267)	*P* Value
Basic Data				
Age (years), M (Q₁, Q₃)	58.00 (50.00, 67.00)	57.00 (48.00, 65.00)	60.00 (52.00, 68.00)	0.003
BMI, M (Q₁, Q₃)	25.90 (23.86, 28.68)	25.82 (23.85, 28.68)	25.97 (23.87, 28.68)	0.756
SBP(mmHg), M (Q₁, Q₃)	127.00 (114.00, 139.00)	128.00 (113.75, 141.00)	126.00 (115.00, 138.00)	0.565
DBP (mmHg), M (Q₁, Q₃)	80.00 (71.00, 86.00)	79.00 (71.00, 86.00)	80.00 (70.50, 86.50)	0.821
Heart rate (bpm), M (Q₁, Q₃)	78.00 (69.50, 86.00)	77.00 (68.75, 85.00)	78.00 (70.00, 86.50)	0.543
KILLIP Grade ≥ 2, *n*(%)	49 (8.89)	27 (9.51)	22 (8.24)	0.601
Male, *n*(%)	474 (86.18)	250 (88.34)	224 (83.90)	0.131
Smoking, *n*(%)	282 (51.18)	147 (51.76)	135 (50.56)	0.778
Length of hospital stay	6.00 (5.00, 7.00)	6.00 (4.00, 7.00)	6.00 (5.00, 7.00)	<.001
Hematological index				
WBC (10*9/L), M (Q₁, Q₃)	10.00 (8.10, 12.10)	9.50 (7.70, 11.50)	10.90 (8.60, 12.65)	<.001
Neu (10*9/L), M (Q₁, Q₃)	7.96 (6.25, 10.25)	7.44 (5.92, 9.59)	8.84 (6.88, 10.65)	<.001
Lym, M (Q₁, Q₃)	1.30 (1.00, 1.80)	1.40 (1.10, 1.80)	1.30 (0.90, 1.75)	0.039
PLT, M (Q₁, Q₃)	210.00 (177.00, 254.00)	213.50 (178.75, 255.25)	209.00 (177.00, 252.00)	0.461
PeakhsCRP, M (Q₁, Q₃)	24.50 (10.75, 58.70)	18.20 (7.70, 42.30)	31.90 (16.40, 69.90)	<.001
LCR, M (Q₁, Q₃)	0.05 (0.02, 0.13)	0.07 (0.03, 0.20)	0.04 (0.02, 0.08)	<.001
PLR, M (Q₁, Q₃)	155.00 (115.11, 221.62)	148.88 (113.88, 206.18)	165.00 (118.97, 230.45)	0.077
NLR, M (Q₁, Q₃)	5.85 (4.02, 8.91)	5.27 (3.77, 7.82)	6.90 (4.47, 10.03)	<.001
Log-Peak hs-TnT (ng/L), M (Q₁, Q₃)	8.15 (7.40, 8.83)	7.68 (7.00, 8.24)	8.68 (8.08, 9.16)	<.001
Log-Peak NT- proBNP (pg/ml), M (Q₁, Q₃)	7.21 (6.66, 7.74)	7.08 (6.52, 7.61)	7.32 (6.82, 7.84)	0.003
TG(mmol/L), M (Q₁, Q₃)	1.39 (0.94, 2.11)	1.45 (1.00, 2.08)	1.32 (0.90, 2.13)	0.328
TC(mmol/L), M (Q₁, Q₃)	4.32 (3.69, 5.03)	4.38 (3.66, 5.01)	4.31 (3.71, 5.03)	0.962
LDL-C(mmol/L), M (Q₁, Q₃)	2.73 (2.15, 3.38)	2.77 (2.19, 3.38)	2.68 (2.12, 3.36)	0.645
HDL-C(mmol/L), M (Q₁, Q₃)	0.92 (0.79, 1.10)	0.92 (0.80, 1.09)	0.93 (0.78, 1.10)	0.950
HbA1c(%), M (Q₁, Q₃)	6.00 (5.60, 7.00)	5.91 (5.60, 7.40)	6.00 (5.60, 6.90)	0.967
eGFR, M (Q₁, Q₃)	113.14 (99.49, 120.00)	115.73 (99.71, 120.00)	110.82 (99.38, 120.00)	0.274
CMR parameters				
CMR examination time, M (Q₁, Q₃)	5.00 (4.00, 6.00)	5.00 (4.00, 6.00)	5.00 (4.00, 6.00)	0.424
LVEF (%), M (Q₁, Q₃)	52.00 (49.96, 56.00)	53.00 (51.00, 57.00)	51.00 (47.27, 53.10)	<.001
LGE%, M (Q₁, Q₃)	27.30 (17.82, 40.41)	21.00 (13.44, 30.34)	34.72 (24.55, 48.31)	<.001
MVO%, M (Q₁, Q₃)	0.00 (0.00, 2.16)	0.00 (0.00, 0.00)	2.34 (1.06, 5.23)	<.001
Past History				
Hypertension, *n*(%)	249 (45.19)	125 (44.01)	124 (46.44)	0.567
Diabetes, *n*(%)	143 (25.95)	83 (29.23)	60 (22.47)	0.071
Stroke, *n*(%)	56 (10.16)	27 (9.51)	29 (10.86)	0.599
Coronary Angiography Results				
Preoperative-TIMI Grade ≤ 1, *n*(%)	446 (80.94)	213 (75.00)	233 (87.27)	<.001
LCX, *n*(%)	69 (12.52)	37 (13.03)	32 (11.99)	0.712
LAD, *n*(%)	254 (46.10)	138 (48.59)	116 (43.45)	0.226
RCA, *n*(%)	228 (41.38)	109 (38.38)	119 (44.57)	0.140
Medication				
Antiplatelets, *n*(%)	544 (98.73)	282 (99.30)	262 (98.13)	0.399
Statins, *n*(%)	531 (96.37)	277 (97.54)	254 (95.13)	0.132
ACEI/ARB, *n*(%)	312 (56.62)	159 (55.99)	153 (57.30)	0.755
β-blockers, *n*(%)	466 (84.57)	237 (83.45)	229 (85.77)	0.452
Spironolactone, *n*(%)	63 (11.45)	24 (8.45)	39 (14.66)	0.022

MVO, microvascular obstruction; BMI, body mass index; SBP, systolic blood pressure; DBP, diastolic blood pressure; bpm, beats per minute; WBC, white blood cell; Neu, neutrophil; Lym, lymphocyte; PLT, platelet; hs-CRP, high-sensitivity C-reactive protein; LCR, lymphocyte to high-sensitivity C-reactive protein ratio; PLR, platelet-to-lymphocyte ratio; NLR, neutrophil-to-lymphocyte ratio; hs-TnT, high-sensitivity cardiac troponin T; NT-proBNP, N-terminal pro-B-type natriuretic peptide; TC, total cholesterol; TG, triglyceride; LDL-C, low-density lipoprotein cholesterol; HDL-C, high-density lipoprotein cholesterol; eGFR, estimated glomerular filtration rate; LVEF, left ventricular ejection fraction; LGE, late gadolinium enhancement; MVO, microvascular obstruction; TIMI, thrombolysis in myocardial infarction; LCX, left circumflex artery; LAD, left anterior descending artery; RCA, right coronary artery; ACEI, angiotensin-converting enzyme inhibitor; ARB, angiotensin II receptor blockers; M, Median, Q₁: 1st Quartile, Q₃: 3st Quartile.

### Correlation between LCR and predictive indicators of MVO

**Figure 3:**
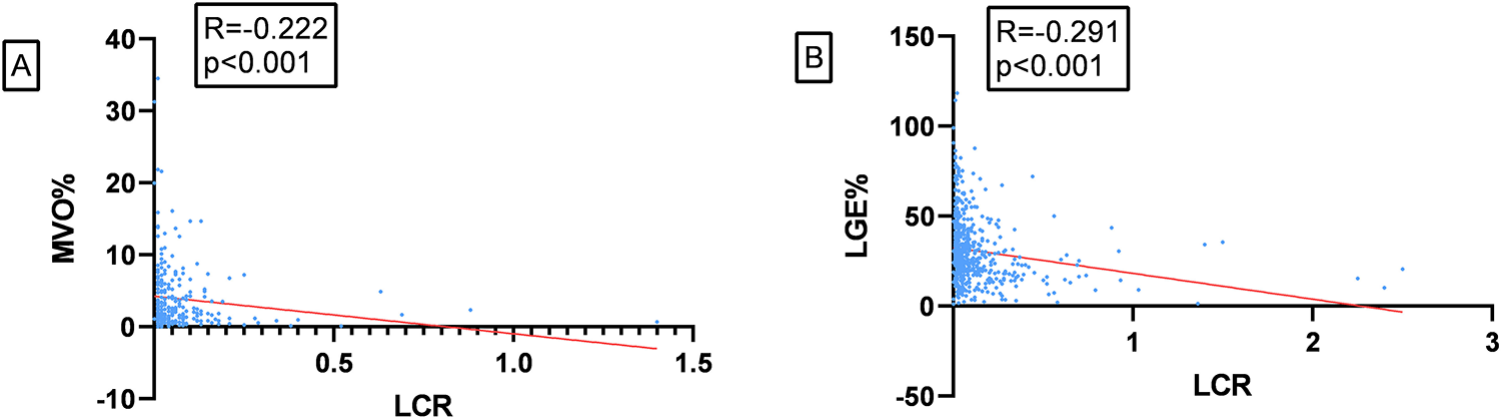
**(A)** correlation between LCR and MVO%. **(B)** Correlation between LCR and LGE%.

There was a linear correlation between LCR and MVO mass: in patients with MVO, Spearman analysis revealed a negative linear correlation between LCR and MVO% (*r* = −0.222, *p* < 0.001, *n* = 267), as well as between LCR and LGE% (*r* = −0.291, *p* < 0.001, *n* = 551), as shown in Figures 3A,B, respectively.

### Logistic regression analysis

Univariate logistic regression analysis (Table 2) showed that MVO was associated with preoperative TIMI flow grade, age, longer hospital stays, peak hs-TnT, peak NT-proBNP, peak hs-CRP, LVEF, LCR, NLR, white blood cell count, neutrophil count, and LGE% (*p* < 0.05). These factors were included in a multivariate stepwise logistic regression analysis (Table 3), which identified peak hs-TnT, peak NT-proBNP, preoperative TIMI flow grade ≤ 1, LCR, and LGE% as independent predictors of MVO.

**Table 2:** Unvariable logistic.

Variables	β	S.E	Z	*P*	OR (95% CI)
Sex	−0.37	0.25	−1.50	0.133	0.69 (0.42–1.12)
Smoking	−0.05	0.17	−0.28	0.778	0.95 (0.68–1.33)
Hypertension	0.10	0.17	0.57	0.567	1.10 (0.79–1.54)
Diabetes	−0.35	0.20	−1.80	0.071	0.70 (0.48–1.03)
Stroke	0.15	0.28	0.53	0.599	1.16 (0.67–2.02)
KILLIP Grade ≥ 2	−0.16	0.30	−0.52	0.602	0.85 (0.47–1.54)
length of hospital stay	0.13	0.04	3.25	0.001	1.14 (1.05–1.23)
Preoperative-TIMI Grade ≤ 1	0.83	0.23	3.61	<.001	2.28 (1.46–3.58)
CMR examination time	−0.01	0.01	−0.58	0.561	0.99 (0.98–1.01)
LCX	−0.10	0.26	−0.37	0.712	0.91 (0.55–1.51)
LAD	−0.21	0.17	−1.21	0.226	0.81 (0.58–1.14)
RCA	0.26	0.17	1.47	0.141	1.29 (0.92–1.81)
Age	0.02	0.01	3.23	0.001	1.02 (1.01–1.04)
BMI	0.01	0.02	0.47	0.638	1.01 (0.97–1.06)
SBP	−0.00	0.00	−0.51	0.613	1.00 (0.99–1.01)
DBP	0.00	0.01	0.04	0.970	1.00 (0.99–1.01)
Heart rate	0.00	0.01	0.71	0.480	1.00 (0.99–1.02)
LCR	−3.78	0.82	−4.63	<.001	0.02 (0.00–0.11)
WBC	0.11	0.03	3.79	<.001	1.12 (1.05–1.18)
Neu	0.11	0.03	3.81	<.001	1.12 (1.06–1.19)
PLT	−0.00	0.00	−0.88	0.379	1.00 (1.00–1.00)
PLR	−0.00	0.00	−0.01	0.989	1.00 (1.00–1.00)
NLR	0.05	0.02	2.71	0.007	1.05 (1.01–1.08)
Log-Peakhs-TnT	1.20	0.12	9.61	<.001	3.32 (2.60–4.24)
Log-PeakNT- proBNP	0.29	0.10	3.00	0.003	1.33 (1.10–1.61)
TG	0.04	0.05	0.73	0.468	1.04 (0.94–1.15)
TC	−0.02	0.08	−0.28	0.777	0.98 (0.84–1.14)
LDL-C	−0.06	0.08	−0.74	0.462	0.94 (0.80–1.11)
HDL-C(	0.22	0.34	0.67	0.503	1.25 (0.65–2.41)
HbA1c(%)	−0.06	0.05	−1.07	0.285	0.94 (0.85–1.05)
eGFR	−0.00	0.01	−0.15	0.877	1.00 (0.99–1.01)
LVEF	−0.08	0.01	−5.31	<.001	0.92 (0.90–0.95)
LGE%	0.06	0.01	9.04	<.001	1.07 (1.05–1.08)
Antiplatelets	−0.99	0.84	−1.18	0.239	0.37 (0.07–1.93)
Statins	−0.71	0.48	−1.48	0.139	0.49 (0.19–1.26)
ACEI/ARB	0.05	0.17	0.31	0.755	1.06 (0.75–1.48)
β-blockers	0.10	0.18	0.55	0.582	1.11 (0.77–1.59)
Spironolactone	0.62	0.27	2.26	0.024	1.86 (1.09–3.19)

TIMI, thrombolysis in myocardial infarction; LCX, left circumflex artery; LAD, left anterior descending artery; RCA, right coronary artery; BMI, body mass index; SBP, systolic blood pressure; DBP, diastolic blood pressure; bpm, beats per minute; WBC, white blood cell; Neu, neutrophil; PLT, platelet; PLR, platelet-to-lymphocyte ratio; NLR, neutrophil-to-lymphocyte ratio; Log-Peak hs-TnT, logarithm of peak of high-sensitivity cardiac troponin T; Log-Peak NT-proBNP, logarithm of peak of N-terminal pro-B-type natriuretic peptide; LCR, lymphocyte to high-sensitivity C-reactive protein ratio; TC, total cholesterol; TG, triglyceride; LDL-C, low-density lipoprotein cholesterol; HDL-C, high-density lipoprotein cholesterol; eGFR, estimated glomerular filtration rate; BNP, B-type natriuretic peptide; LVEF, left ventricular ejection fraction; LGE%, late gadolinium enhancement; ACEI, angiotensin-converting enzyme inhibitor; ARB, angiotensin II receptor blockers. OR: odds ratio, CI: confidence interval.

**Table 3:** Multivariable logistic.

Variables	β	S.E	Z	P	OR (95% CI)
Preoperative-TIMI Grade ≤ 1	0.55	0.27	2.03	0.043	1.73 (1.02–2.95)
LCR	−1.72	0.73	−2.35	0.019	0.18 (0.04–0.75)
Peak hs-TnT	0.84	0.14	6.11	<.001	2.31 (1.77–3.02)
Peak NT- proBNP	−0.25	0.12	−2.09	0.036	0.78 (0.61–0.98)
LGE%	0.05	0.01	5.79	<.001	1.05 (1.03–1.06)

TIMI, thrombolysis in myocardial infarction; LCR, lymphocyte to high-sensitivity C-reactive protein ratio; hs-TnT, high-sensitivity cardiac troponin T; NT-proBNP, N-terminal pro-B-type natriuretic peptide; LGE, late gadolinium enhancement; OR, odds ratio; CI, confidence interval.

**Figure 4:**
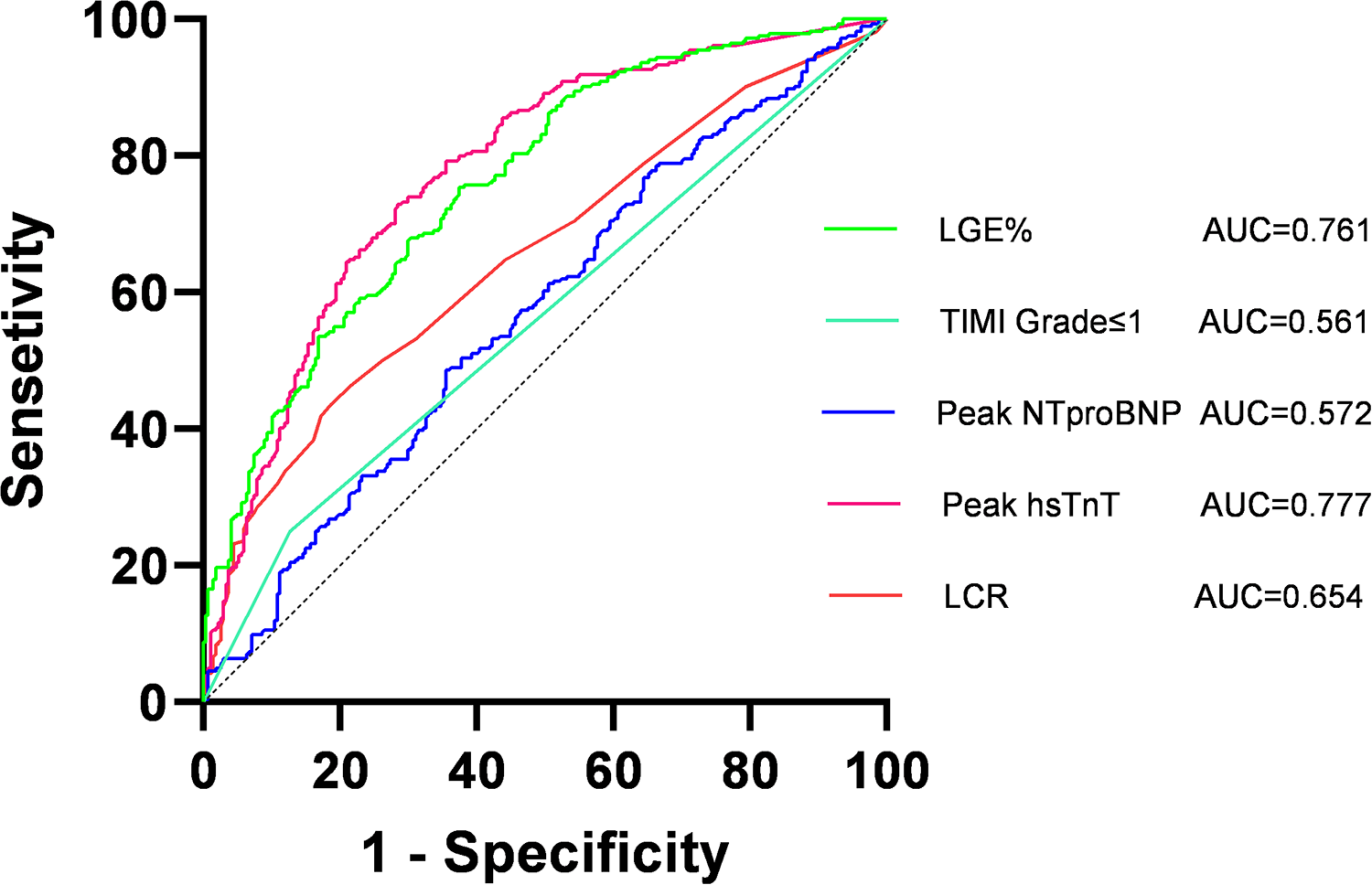
Receiver operating characteristic (ROC) curves of lymphocyte to high-sensitivity C-reactive protein ratio (LCR), thrombolysis in myocardial infarction (TIMI); late gadolinium enhancement (LGE%). AUC, area under curve.

**Figure 5:**
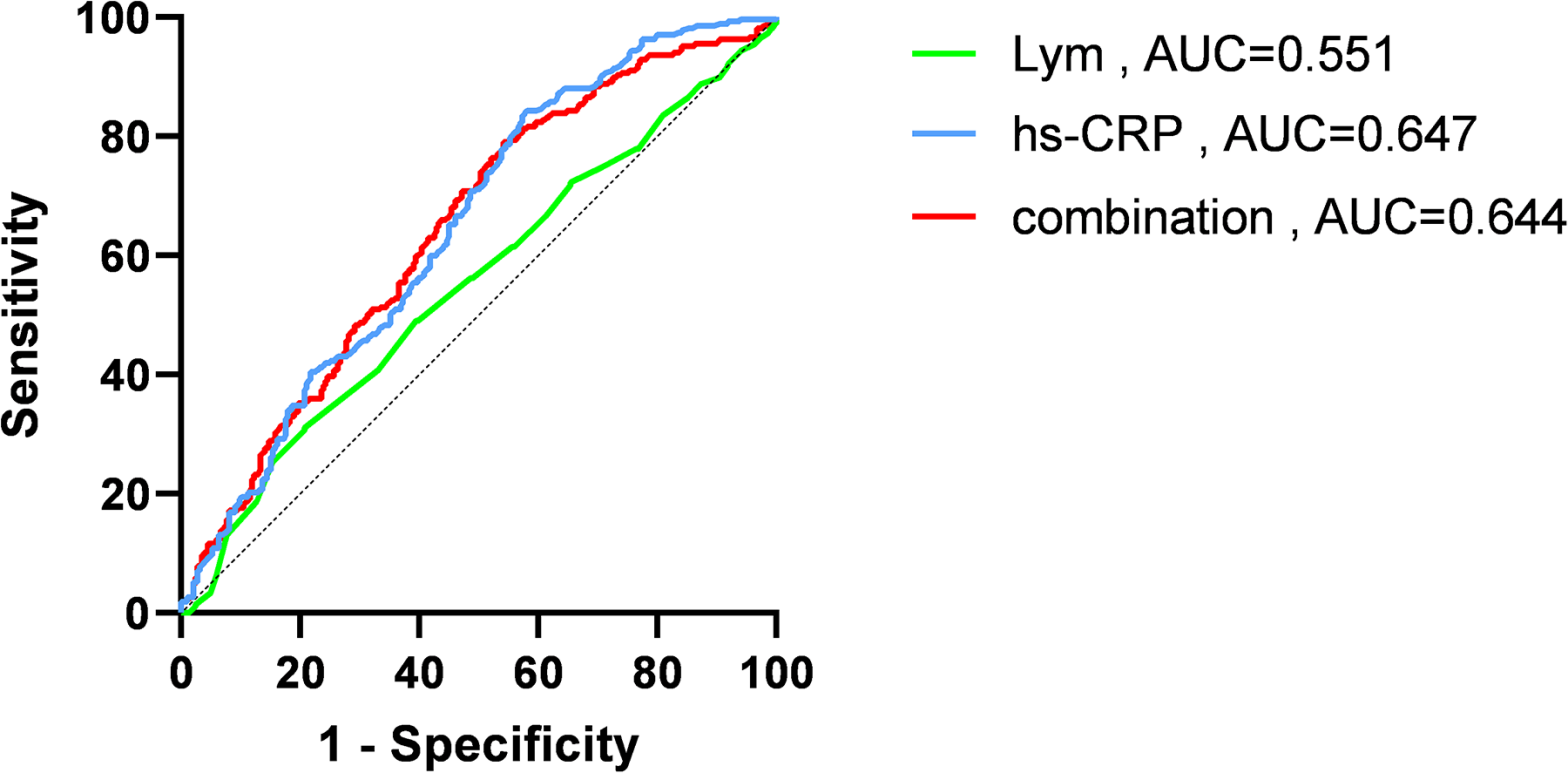
Receiver operating characteristic (ROC) curves of high-sensitivity C-reactive protein (hs-CRP), lymphocyte (Lym) and combination of the two. AUC, area under curve.

ROC curve analysis of the five factors identified in the multivariate regression analysis (Figure 4 and Table 4) revealed that peak NT-proBNP [Area under the Curve (AUC) = 0.572, cut-off: 1,123.5, 95% Confidence Interval (CI): 0.524–0.620, *p* < 0.001], preoperative TIMI flow grade ≤ 1 (AUC = 0.561, 95% CI: 0.514–0.609, *p* < 0.001), peak hs-TnT (AUC = 0.777, cut-off: 3,525, 95% CI: 0.738–0.816, *p* < 0.001), LCR (AUC = 0.654, cut-off: 0.091, 95% CI: 0.609–0.700, *p* < 0.001), and LGE% (AUC = 0.761, cut-off: 30.4, 95% CI: 0.721–0.800, *p* < 0.001) all had predictive value for MVO occurrence. We also plotted ROC curves for peak CRP, lymphocyte counts, and their combination (Figure 5).

**Table 4:** ROC curve analysis.

Variables	AUC	95% CI	P	Cut-off	Sensitivity	Specificity
Peak NT-proBNP	0.572	0.524–0.620	<0.001	1,123.5	0.644	0.486
Peak hs-TnT	0.777	0.738–0.816	<0.001	3,525	0.719	0.725
Preoperative-TIMI Grade ≤ 1	0.561	0.514–0.609	<0.001	-	0.873	0.250
LGE%	0.761	0.721–0.800	<0.001	30.41	0.625	0.754
LCR	0.654	0.609–0.700	<0.001	0.091	0.801	0.454

NT-proBNP, N-terminal pro-B-type natriuretic peptide; hs-TnT, high-sensitivity cardiac troponin T; TIMI, thrombolysis in myocardial infarction; LGE, late gadolinium enhancement; LCR, lymphocyte to high-sensitivity C-reactive protein ratio; CI, confidence interval; AUC, area under curve.

### Prediction model analysis

**Figure 6:**
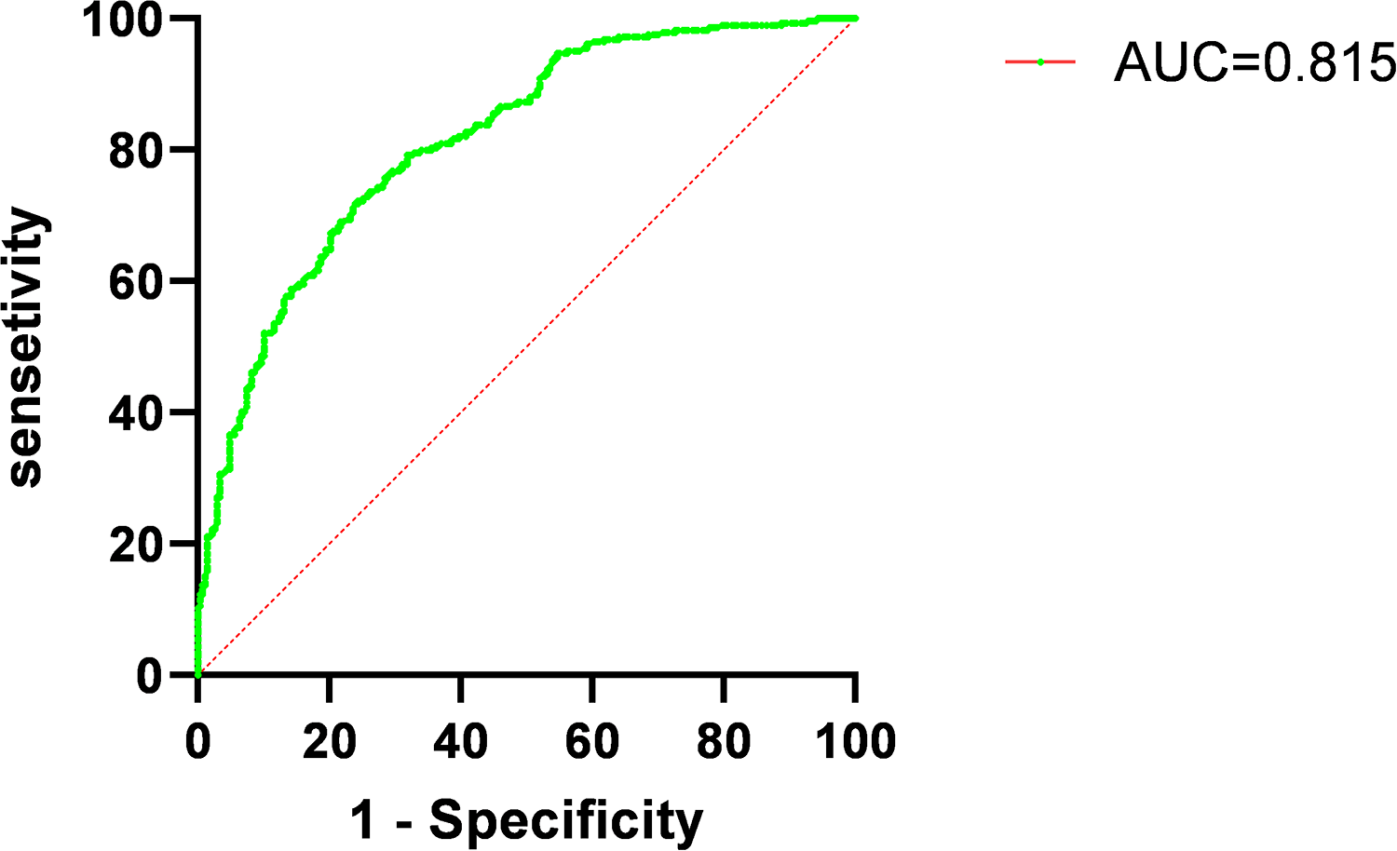
Receiver operating characteristic curves (ROC) for combined measures predicting occurrence of microvascular obstruction (MVO) in patients with acute ST-elevation myocardial infarction (STEMI). AUC, area under curve.

A new predictive model incorporating these five independent risk factors was developed, and ROC curve analysis showed good predictive performance (Figure 6, **AUC = 0.815, 95% CI: 0.78–0.85, *p*** **<** **0.001**). NRI (*p* = 0.004) and IDI (*p* = 0.023) analyses showed significant differences between the new and old predictive models (Table 5). The NRI value of 0.225 > 0 indicates that the new model outperformed the old one, while the IDI value of 0.01 > 0 indicates that the new predictive model provided a positive improvement over the old one.

**Table 5:** Discrimination accuracy and reclassification of LCR for MVO.

Model	NRI		IDI	
	Estimate (95% CI)	*P* value	Estimate (95% CI)	*P* value
Conventional model^a^	Reference	-	Reference	-
Conventional model + LCR	0.225 (0.0737–0.3759)	0.004	0.01 (0.0014–0.0185）	0.023

LCR, lymphocyte to high-sensitivity C-reactive protein ratio.

^a^ Conventional model included Logarithm of peak of high-sensitivity cardiac troponin T, Logarithm of peak of N-terminal pro-B-type natriuretic peptide, Late Gadolinium Enhancement, Preoperative- thrombolysis in myocardial infarction Grade.

## Discussion

To our knowledge, this is the first study to explore the relationship between LCR and MVO in patients with STEMI. The main findings of our study are as follows:

1. There was a significant difference in LCR between patients with and without MVO during hospitalization.
2. LCR showed a significant negative linear correlation with MVO%.
3. After adjusting for confounding factors, including LVEF, low LCR was found to be an independent risk factor for MVO in patients with STEMI.
4. The predictive model incorporating LCR improved the ability to predict MVO occurrence post-PCI.

As a non-invasive gold standard for cardiac assessment, CMR effectively identifies myocardial infarct size and MVO regions in patients with STEMI. Despite successful reperfusion of major vessels with PCI, approximately 50% of patients still experience MVO (2). In our study, 267 patients (48.5%) developed MVO. Current research indicates that MVO arises from distal embolization caused by thrombi and plaque fragments, microvascular spasms, endothelial dysfunction, direct myocardial injury, and edema caused by ischemia-reperfusion injury. Studies have suggested that microvascular dysfunction and myocardial hemorrhage can lead to the deposition of iron crystals in infarcted myocardial regions, further inducing an inflammatory response (13). Ischemia-reperfusion injury can also lead to apoptosis of myocardial and endothelial cells, while neutrophil-platelet aggregates can obstruct micro-vessels and release vasoconstrictors and inflammatory mediators (14). Distal embolization also causes myocardial contractile dysfunction, promoting MVO (15).

The age-thrombus burden-microvascular resistance index score effectively identifies microvascular injury and predicts MVO (16). A lower LVEF suggests impaired cardiac pumping ability, which affects microcirculatory blood flow and is closely related to MVO (17). Plasma troponin levels are also associated with the presence and severity of MVO after STEMI reperfusion and have greater predictive value than electrocardiogram scoring parameters (18, 19). Elevated NT-proBNP levels are similarly linked to MVO, while larger infarct size and lower initial TIMI flow grades are also correlated with MVO, which was confirmed in our study (20–22).

Inflammatory responses are central to the entire myocardial infarction process, and patients with elevated inflammatory markers are at higher risk for STEMI and its complications (23). Larger infarcts induce stronger inflammatory responses, with local and systemic inflammation contributing to plaque instability and reduced myocardial perfusion, further aggravating myocardial damage. Studies have shown that residual inflammatory risk assessed by hs-CRP levels better predicts future cardiovascular events and mortality than residual cholesterol risk assessed by LDL-C (24). There is growing evidence that hs-CRP promotes myocardial necrosis and accumulates in infarct regions (25). It also affects endothelial fibrinolytic balance, promoting fibrin formation and coagulation, and activating complement to induce thrombosis (26). A prospective study found that CRP velocity in patients with STEMI undergoing PCI is an independent predictor of MVO as assessed by CMR (27). Lymphocytes, on the other hand, help maintain atherosclerotic plaque stability (28). During myocardial infarction, elevated catecholamine and cortisol levels increase lymphocyte apoptosis, which may partly explain the inverse relationship between peripheral lymphocyte count and cardiovascular risk (29). Particularly T-lymphocytes, play an essential role in the resolution of inflammation and tissue repair. A decrease in lymphocyte count, suggests impaired immune regulation and a reduced capacity for tissue recovery post-injury (30). By combining these two indicators, namely lymphocyte count and CRP levels, LCR reflects both inflammation and immune status during myocardial infarction, and better predicts adverse events in post-PCI patients with myocardial infarction compared to lymphocyte counts or CRP levels alone (7). Low LCR may reflect an imbalance between pro-inflammatory cytokines and anti-inflammatory mediators. The increased CRP levels seen in low LCR contribute to a sustained inflammatory state, while the relative decrease in lymphocytes may indicate a suppressed adaptive immune response (31). This imbalance may worsen endothelial dysfunction and promote the development of MVO, further exacerbating the ischemic damage in STEMI patients (32, 33).

Our study has innovatively identified LCR as an independent risk factor for MVO. We hypothesize that inflammation exacerbates myocardial apoptosis and interstitial edema, compressing capillaries and small arteries, leading to microvascular dysfunction and MVO. Previous studies have shown that MVO is related to infarct size, as myocardial necrosis stimulates pro-inflammatory responses (21, 34). In our study, LCR was significantly negatively correlated with infarct size (LGE%), further supporting our findings.

We improved traditional predictive models by incorporating LCR as a new variable, enhancing the predictive capability of our new model. The new model statistically improves risk prediction accuracy and provides important clinical guidance. This improvement allows for more effective patient risk stratification, enabling the development of more refined treatment plans for high-risk patients, ensuring closer monitoring and intervention. The predictive information of the new model can also guide clinical strategies to reduce MVO risk and improve patient outcomes and quality of life.

Our study shows that lower LCR is significantly associated with higher MVO risk, suggesting that inflammatory activity may promote microvascular dysfunction. As a non-invasive biomarker, LCR helps assess the inflammatory response of the patient and the associated MVO risk. In conclusion, this study revealed a significant correlation between LCR and MVO in patients with STEMI and emphasizes the importance of inflammation in microvascular dysfunction. Monitoring LCR changes may allow timely adjustment of treatment plans to mitigate MVO occurrence. With further research, LCR is expected to become a key biomarker for guiding the treatment of patients with STEMI. Incorporating LCR measurements into routine clinical assessment and using it alongside other established markers to improve patient management and decision-making.

## Limitations

Although the current study provides preliminary insights into the relationship between LCR and MVO, further research is needed to determine the specific mechanisms and clinical applications of this association. Our study was a single-center, retrospective investigation with a relatively small sample size and it may be subject to selection bias. Future studies should adopt multi-center, large-scale, randomized controlled designs to validate the effectiveness of LCR as a predictive tool, improve external validity and examine its variability across different treatment strategies. Additionally, this study only explored the relationship between LCR and MVO in patients with STEMI, so the findings cannot be generalized to other patient cohorts with various other diseases. Furthermore, as a retrospective study, our research is a correlation analysis and cannot establish causal relationships. Lastly, while lower LCR was associated with a higher incidence of MVO, we did not investigate whether anti-inflammatory treatment could reduce the risk of MVO in these patients.

## Conclusion

Low LCR is an independent risk factor for MVO in patients with STEMI after PCI. Incorporating LCR into predictive models improves the ability to forecast MVO occurrence in these patients.

## Data Availability

The raw data supporting the conclusions of this article will be made available by the authors, without undue reservation.
